# A Source Localization Method Using Complex Variational Mode Decomposition

**DOI:** 10.3390/s22114029

**Published:** 2022-05-26

**Authors:** Qiuyan Miao, Xinglin Sun, Bin Wu, Lingyun Ye, Kaichen Song

**Affiliations:** College of Biomedical Engineering and Instrument Science, Zhejiang University, Hangzhou 310027, China; 11715040@zju.edu.cn (Q.M.); wubinzju@zju.edu.cn (B.W.); lyye@zju.edu.cn (L.Y.); kcsong@zju.edu.cn (K.S.)

**Keywords:** source localization, complex variational mode decomposition, near-field source, far-field source, compressive sensing

## Abstract

Source localization with a passive sensors array is a common topic in various areas. Among the popular source localization algorithms, the compressive sensing (CS)-based method has recently drawn considerable interest because it is a high-resolution method, robust with coherent sources and few snapshots, and applicable for mixed near-field and far-field source localization. However, the CS-based methods rely on the dense grid to ensure the required estimation precision, which is time-consuming and impractical. This paper applies the complex variational mode decomposition (CVMD) to source localization. Specifically, the signal model of the source localization problem is similar to the time-domain frequency-modulated signal model. Motivated by this, we extend CVMD, initially designed for nonstationary time-domain signal analysis, to array signal processing. The decomposition results of the array measurements can correspond to the potential sources at different locations. Then, the sources’ direction and range can be estimated by model fitting with the decomposed subsignals. The simulation results show that the proposed CVMD-based method can locate the pure far-field, pure near-field, mixed far-field, and near-field sources. Notably, it can yield high-resolution localization for the coherent sources with one single snapshot with low computing time.

## 1. Introduction

Source localization is a common topic in various areas [1,2]. A variety of positioning methods for different applications have been extensively studied recently. For example, the autonomous driving system uses active sources to locate the vehicle [3]; radio Frequency technologies are engaged for indoor localization [4]; a virtual array by moving unmanned aerial vehicles is applied to wireless communication systems [5]. However, for some applications, such as sonar, radar, and speaker localization, utilizing a passive sensor array is the most typical positioning way. Therefore, there is great interest in passive sensor array signal processing for achieving excellent positioning performance [6]. According to the distance between the array and the source, most localization algorithms divide the problem into far-field and near-field scenarios [7]. These two cases have each been investigated intensively for decades [8,9,10].

For the far-field scenario, the array signal processing methods often focus on the direction of the arrival (DOA) estimation problem. Among the DOA estimation approaches, conventional beamforming (CBF) [11], also known as the delay and sum algorithm, is the most common and robust. However, it is limited by the Rayleigh threshold, resulting in low resolution and high-level sidelobes, making it challenging to distinguish adjacent sources and detect weak targets [12]. Consequently, several high-resolution beamforming approaches have been presented, including the minimum variance distortion-free response (MVDR) [13], the multiple signal classification (MUSIC) [14], and the estimation of signal parameters via rotational invariance techniques (ESPRIT) [15]. Although the mentioned approaches have narrow beamwidths and low sidelobe levels, their performance depends on the accurate estimation of the data covariance matrix. Therefore, they require many samples of signals from sensors to compute the covariance matrix [16]. Simultaneously, these methods are sensitive to signal model mismatches and need the sources to be uncorrelated. Recently, some novel methods based on sparse signal recovery have been proved to outperform the above techniques in terms of resolution and robustness, which is also suitable for a few snapshots and correlated sources [17,18]. The compressive sensing (CS) beamforming method is typical [19]. However, these methods often rely on the dense grid to guarantee the required estimation precision, which is time-consuming.

For the near-field scenario, both DOA and source range need to be jointly estimated. Some far-field DOA estimation methods are extended to locate near-field sources, such as MUSIC-based [20] and ESPRIT-based methods [21]. These approaches and their variants have the same limitations as the far-field DOA estimation; they need the sources to be uncorrelated and require many snapshots. On the other hand, some time-frequency techniques, such as Wigner–Ville distributed method [22] and the discrete fractional Fourier transform method [23], are engaged in near-field source parameter estimation. They can deal with one snapshot, coherent source, and mixed near-field and far-field sources conditions. However, these algorithms cannot meet the requirements of high resolution. With the sparse signal recovery-based methods showing outstanding performance in far-field localization, significant interest has been driven to apply sparse signal recovery-based methods to near-field localization [24]. However, these methods need to generate a grid not only for the possible DOAs but also for the possible ranges, which costs higher computation than that in DOA estimation. Moreover, suppose the source position is not precisely located on the grid, which is common in practical applications. In that case, a basis mismatch will occur, and these grid-based methods’ performance will degrade [25,26].

In summary, the challenges in source localization can be classified into the following aspects. On the one hand, we want the approach to be a robust high-resolution approach with coherent sources and few snapshots and applicable for both near-field and far-field localization. On the other hand, we desire the algorithm to have low calculation. These requirements are common in practice. Therefore, it is vital to propose a source localization method to solve these problems.

From the methods mentioned above, we can draw the following two conclusions. Firstly, sparse signal recovery-based methods have many advantages except for the high computational cost. Secondly, the time-frequency techniques can deal with the mixed near-field and far-field sources on the condition of one snapshot and correlated sources, but it is limited to the resolution. Motivated by this, we use a novel sparse time-frequency representation technique named variational mode decomposition (VMD) to source the localization. VMD is a nonstationary signal analysis method that can adaptively decompose a multicomponent real-valued signal into several subsignals [27,28]. Since array signals are always complex-valued for performing the phase of the different array elements, standard VMD cannot be applied directly. Recently, a complex VMD (CVMD) method was proposed [29], which provides the opportunity for applying VMD to array signal processing. In particular, we have proposed a modified CVMD method in [30], which gives a natural extension for VMD to the complex domain. This paper applies the modified CVMD in far-field and near-filed sources localization, aiming to yield great performance with small computation.

The rest of this paper is organized as follows. Firstly, the signal model is introduced in Section 2. Section 3 reviews the popular existing CS-based method and presents the CVMD method, which will be applied in the proposed algorithm. Section 4 further formulates the problem and proposes the CVMD-based source localization method. Then, we validate our CVMD-based algorithm in various conditions in Section 5. At last, the conclusion is made in Section 6.

## 2. Signal Model

**Figure 1:**
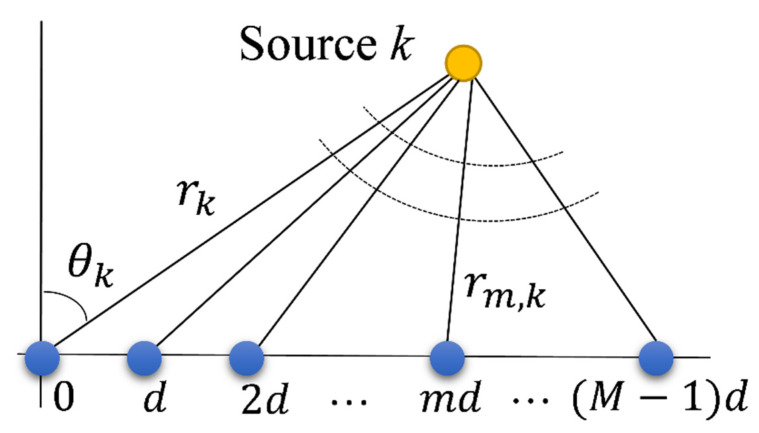
Signal model.

Suppose M sensor elements space in a uniform linear array (ULA), as shown in Figure 1. The interelement spacing is d. Assume that K (near-field or far-field) narrowband signal sources impinge on the ULA. The signal received by the *m*-th (m=0,1,…,M−1) sensor at one snapshot can be expressed as

(1) $$y_{m}=\overset{K}{\underset{k=1}{\sum }}s_{k}\exp\left(\frac{j2\pi }{\lambda }\left(r_{m,k}-r_{k}\right)\right)+n_{m},$$

where

(2) $$r_{m,k}=\sqrt{r_{k}^{2}+m^{2}d^{2}-2mdr_{k}\sin\left(\theta_{k}\right)},$$

represents the distance between the *m*-th sensor and the *k*-th source, $r_{k}$ is the range from the *k*-th source to the zeroth sensor, which is set as a phase reference, $s_{k}$ is the signal radiated by the *k*-th source and received by the zeroth sensor, λ denotes the wavelength of the source signal, and $n_{m}$ means the additive noise. In this paper, we suppose the array element spacing *d* is 1/2 of the wavelength λ.

In most sources’ localization approaches, the phase difference between the *m*-th sensor and the zeroth sensor is simplified as two terms of Taylor expansion as

(3) $$\varphi_{mk}=\frac{2\pi }{\lambda }\left(\sqrt{r_{k}^{2}+m^{2}d^{2}-2mdr_{k}\sin\left(\theta_{k}\right)}-r_{k}\right)\approx -\frac{2\pi dm}{\lambda }\sin\theta_{k}+\frac{\pi d^{2}m^{2}}{\lambda r_{k}}\cos^{2}\theta_{k}.$$

When the range $r_{k}\in \left[0.62{\left(\frac{D^{3}}{\lambda }\right)}^{\frac{1}{2}},\frac{2D^{2}}{\lambda }\right]$, and D=Md denotes array aperture, the *k*-th source is regarded as a near-field source. When the range $r_{k}$ is beyond $\frac{2D^{2}}{\lambda }$, the source is regarded as a far-field source. For the far-field sources, the phase difference $\varphi_{mk}$ can be further simplified as

(4) $$\varphi_{mk}\approx -\frac{2\pi dm}{\lambda }\sin\theta_{k}.$$

Therefore, only the source direction $\theta_{k}$ needs to be estimated in the far-field scene.

In our proposed method, we do not make any simplification of the signal model. The exact spatial propagation geometry in Figure 1 and the exact expression as Equations (1) and (2) are utilized for both far-field and near-field sources localization. Specifically, we use the same model for near-field and far-field scenes. The only difference between the near-field and the far-field source is that both range and direction are required to be estimated in the near-field scenario, while only DOA needs to be solved in far-field sources localization.

## 3. Related Work and Background

### 3.1. Related Work

Existing approaches for source localization with passive sensor array can be divided into three categories. One is the traditional beamforming method based on the Fourier theory, such as CBF. These methods have a limitation on resolution. The second type is the high-resolution algorithm based on the covariance matrix, such as MUSIC and MVDR. These methods cannot deal with the correlated sources. The last category is based on the sparse signal processing theory. These algorithms have advantages in resolution, robustness, and many other aspects. Especially the CS-based localization approach is considered the most representative because of its widespread use and good acceptance in the community. Hence, we introduce the CS-based method and mainly compare it with our proposed method.

The source localization problem in Section 2 can be regarded as an inverse problem, which can be solved by the existing CS method. Equation (1) can be rewritten as

(5) y=As+n,

where $A=\left[a_{1},a_{2},\ldots ,a_{K}\right]\epsilon {∁}^{M\times K}$ denotes the array manifold, $a_{k}\epsilon {∁}^{M\times 1}$ is the so-called steering, $s={\left[s_{1},\ldots ,s_{k}\right]}^{T}\epsilon {∁}^{K\times 1}$ is the source signals, and n is the additive noise matrix.

For the near-filed or the mixed near and far-field cases, the $a_{k}$ can be described as

(6) $$a\left(\theta_{k},r_{k}\right)=\exp\left(j\frac{2\pi }{\lambda }\left(\sqrt{r_{k}^{2}+m^{2}d^{2}-2mdr_{k}\sin\left(\theta_{k}\right)}-r_{k}\right)\right),$$

where m=0,1,…,M−1. Accordingly, the problem can be described as follows: given the measurements y, the unknown parameters $\theta_{k}$ and $r_{k}$, where k=1,…,K needs to be estimated. That is a typical inverse problem. The existing CS-based algorithm uses sparse reconstruction to solve the inverse problem. Firstly, a grid is made by every possible pair of range and theta. Suppose the *k*-th pair of range and theta is marked as $\bar{\theta_{k}}$ and $\bar{r_{k}}$. A known matrix with steering vectors corresponding to each potential source location is then constructed as

(7) $$A\left(\bar{\theta },\bar{r}\right)=\left[\bar{a_{1}},\bar{a_{2}},\ldots ,\bar{a_{N}}\right]\epsilon {∁}^{M\times N},$$

where N denotes the grid size, $\bar{a_{k}}=a\left(\bar{\theta_{k}},\bar{r_{k}}\right)$, and k=1,2,…,N. Then the inverse problem can be solved by

(8) $$\hat{s}=\underset{s\epsilon {∁}^{N\times 1}}{\arg\;\min}\|y-A(\bar{\theta ,}\bar{r})s{\|}_{2}^{2}+\mu \|s{\|}_{1},$$

where μ is the sparsity regulating parameter, and $\hat{s}\epsilon \;{∁}^{N\times 1}$ represents the signal amplitude related to the possible DOAs. Due to the sparse hypothesis, most of the values in $\hat{s}$ are zero.

Although the near-field model suits both near-field and far-field sources, the CS-based method often uses the simplified model for pure far-field source localization because of the smaller computation. For the far-filed scene, the $a_{k}$ can be described as

(9) $$a\left(\theta_{k}\right)=\exp\left(-\frac{j2\pi dm}{\lambda }\sin\theta_{k}\right),$$

where m=0,1,…,M−1. The grid is made by every possible theta. The known matrix with steering vectors corresponding to each potential source direction as its columns can be described as

(10) $$A\left(\bar{\theta }\right)=\left[a\left(\bar{\theta_{1}}\right),a\left(\bar{\theta_{2}}\right),\ldots ,a\left(\bar{\theta_{N}}\right)\right]\epsilon {∁}^{M\times N}.$$

Similar to the near-filed case, the inverse problem can be solved by

(11) $$\hat{s}=\underset{s\epsilon {∁}^{N\times 1}}{\arg\;\min}\|y-A(\bar{\theta })s{\|}_{2}^{2}+\mu \|s{\|}_{1}.$$

Here, $\hat{s}$ represents the signal amplitude related to the possible DOAs, where most values are zero.

The CS-based method can achieve outstanding performance in resolution and robustness. Nevertheless, it requires generating a dense grid to assure the needed estimation precision, which is time-consuming.

### 3.2. Complex Variational Mode Decomposition

This paper plans to use CVMD to solve the above inverse problem. Here, we give a brief introduction to the VMD and CVMD methods. VMD is a novel solution to inverse problems [23], often used in analyzing nonstationary time signals, and CVMD is the extension of VMD to complex-valued data [26].

Suppose we have a multicomponent real-valued signal series x(t). The VMD method can decompose the signal into a set of subsignals called band-limited intrinsic mode functions (IMFs). If we set $x\left(t\right)=6t^{2}+\cos\left(10\pi t+10\pi t^{2}\right)+0.5\sin\left(40\pi t\right)$ and use VMD to it, three subsignals $6t^{2}$, $\cos\left(10\pi t+10\pi t^{2}\right),$ and 0.5sin(40πt) can be separated and recovered.

Specifically, the VMD method is implemented by the constrained variational problem:

(12) $$\underset{\left\{u_{k}\right\},\left\{\omega_{k}\right\}}{\min}\{\sum_{k=1}^{K}\|\partial_{t}\left[\left(\delta \left(t\right)+\frac{j}{\pi t}\right)*u_{k}\left(t\right)\right]e^{-j\omega_{k}t}{\|}_{2}^{2}\},\;\mathrm{subject}\;\mathrm{to}\;\sum_{k=1}^{K}u_{k}\left(t\right)=x\left(t\right).$$

where the sign ∗ denotes the convolution operation, $\{u_{k}\left(t\right)\}=\left\{u_{1}\left(t\right),\ldots ,u_{K}\left(t\right)\right\}$ are IMFs to be solved, $\{\omega_{k}\}=\left\{\omega_{1},\ldots ,\omega_{K}\right\}$ denotes the center frequencies of the IMFs, and the decomposition number K is supposed to know as a priori.

When it comes to a complex-valued sequence, the standard VMD method is not applicable. CVMD gives a preprocessing to the complex-valued data, then the standard VMD can be applied, and finally, the complex-valued IMFs are obtained. Take the multicomponent complex-valued signal series $x\left(t\right)=0.5e^{j2\pi \left(-8t\right)}+e^{j2\pi \left(-t+0.6t^{2}\right)}+0.8e^{j2\pi \left(16t+0.2t^{2}\right)}$ for example, three complex-valued subsignals $0.5e^{j2\pi \left(-8t\right)}$, $e^{j2\pi \left(-t+0.6t^{2}\right)}$, and $0.8e^{j2\pi \left(16t+0.2t^{2}\right)}$ can be separated and recovered using CVMD.

## 4. Near-Field and Far-Field Sources Localization Algorithm

### 4.1. Problem Formulation

Though the VMD and CVMD are often applied in analyzing time-domain signals, we extend them to the array signal processing. To further establish the contact between CVMD methods to the source localization topic, we rewrite Equation (1) as

(13) $$y\left(m\right)=\overset{K}{\underset{k=1}{\sum }}s_{k}\exp\left(j\varphi_{m}\left(\theta_{k},r_{k}\right)\right)+n_{m},$$

where m=0,1,…,M−1. It can be seen that the measurement y(m) is a multicomponent complex-valued signal, where the sensor index m is similar to the time index in the time domain. Thus, the signal model can be analyzed like the time series.

According to the signal model in Section 2, the $s_{k}\exp\left(j\varphi_{m}\left(\theta_{k},r_{k}\right)\right)$ part, which represents the *k*-th source received by the sensor array, is similar to the frequency-modulated signal model. Moreover, its instantaneous frequency f(m) is monotonically increasing with the index m increasing. Suppose the array elements spacing d is half of the signal wavelength λ. Then the spatial frequency range is between $f\left(0\right)=-\sin(\theta_{k})$ to $f\left(M-1\right)=\frac{\left(D-r_{k}\sin(\theta_{k}\right))}{\sqrt{\left(r_{k}^{2}+D^{2}-2Dr_{k}\sin(\theta_{k})\right)}}$, where D=(M−1)d denotes the array aperture. Consequently, the bandwidth of the signal is $\frac{\left(D-r_{k}\sin(\theta_{k}\right))}{\sqrt{\left(r_{k}^{2}+D^{2}-2Dr_{k}\sin(\theta_{k})\right)}}+\sin(\theta_{k})$.

Since the frequency-modulated signal $s_{k}\exp\left(j\varphi_{m}\left(\theta_{k},r_{k}\right)\right)$ is band-limited, it can be regarded as a subsignal named band-limited complex IMF in the CVMD algorithm. Thus, the inverse problem for the source localization has a different solution. We can use the CVMD method to decompose the measurements y(m) to obtain the possible subsignal $s_{k}\exp\left(j\varphi_{m}\left(\theta_{k},r_{k}\right)\right)$. After decomposition, the parameters $\theta_{k}$ and $r_{k}$ can be estimated by fitting the phase of the IMF with the function $\varphi_{m}\left(\theta_{k},r_{k}\right)$. The signal amplitude $s_{k}$ can be obtained by calculating the amplitude of the IMF. In brief, the proposed CVMD-based source localization algorithm has two processes: decomposition and model fitting.

### 4.2. Decomposition

To decompose the array measurements y(m) into several IMFs, we use the modified CVMD method. The decomposition process in modified CVMD contains several steps: signal extension, upsampling, modulation, standard VMD, demodulation, and downsampling. The first three steps can be regarded as the signal preprocessing procedure, aiming to generate the real-valued data from the original complex-valued data for the standard VMD. The last two steps, demodulation and downsampling, are applied to remove the modulation and upsampling and finally obtain the complex-valued decomposition result of the original data.

Suppose we have the measurements by M sensors at one snapshot as the signal model in Section 2, described as y(m), where m=0,1,…,M−1. Firstly, we upsample the measurements y(m) through sinc interpolation to increase the sampling rate. Before interpolation, y(m) are extended by mirroring the signal by half the length on each side. This operation is to reduce the boundary effect of the sinc interpolation. Then the discrete sinc interpolation algorithm is used to double sample the data. Next, the mirror extension part is removed. Finally, the upsampled result $y_{u}\left(m\right)$, where 0≤m≤2M−1, is obtained.

For the original measurements, the spatial sampling rate, defined as $\frac{\lambda }{d}$, is 2. After double sampling, the spatial sampling rate of $y_{u}\left(m\right)$ becomes 4. $y_{u}\left(m\right)$ is then shifted by multiplying exponential factors $e^{j2\pi \frac{m}{4}}$ as follows

(14) $$y_{us}\left(m\right)=y_{u}\left(m\right)e^{j2\pi \frac{m}{4}},0\leq m\leq 2M-1.$$

The modulated result $y_{us}\left(m\right)$ is now an analytical signal. A real-valued signal can be deduced without losing any information from the original analytical signal as

(15) $$y_{usr}\left(m\right)=R\left[y_{us}\left(m\right)\right],$$

where R[·] denotes taking the real part of the complex-valued data.

Since $y_{usr}\left(m\right)$ is real-valued, the VMD method can be used directly through the constrained variational problem as Equation (12). To be specific, VMD solves the problem in the spectral domain. Suppose the discrete Fourier transform (DFT) of the *k*-th IMF is expressed as ${\hat{u}}_{k}\left(\omega \right)$ and the $y_{usr}\left(m\right)$ in the spectral domain is described as Y(ω). The problem is solved by updating the ${\hat{u}}_{k}\left(\omega \right)$ for all ω>0 until convergence, as follows

(16) $${\hat{u}}_{k}^{n+1}\left(\omega \right)=\frac{Y\left(\omega \right)-\sum_{i<k}{\hat{u}}_{i}^{n+1}\left(\omega \right)-\sum_{i>k}{\hat{u}}_{i}^{n}\left(\omega \right)+\frac{\hat{\lambda }\left(\omega \right)}{2}}{1+2\alpha {\left(\omega -\omega_{k}\right)}^{2}},$$

where *n* denotes the *n*-th iteration, $\hat{\lambda }\left(\omega \right)$ denotes the DFT of Lagrangian multipliers, α represents the balancing parameter of the data-fidelity constraint, and the $\omega_{k}$ is calculated at the center of gravity of the corresponding mode’s power spectrum as

(17) $$\omega_{k}^{n+1}=\frac{\int_{0}^{\infty }\omega {\left|{\hat{u}}_{k}\left(\omega \right)\right|}^{2}d\omega }{\int_{0}^{\infty }{\left|{\hat{u}}_{k}\left(\omega \right)\right|}^{2}d\omega }.$$

Then, the IMFs $\left\{u_{k}\left(m\right)\right\}$ are obtained from the real part of the inverse DFT of the final ${\hat{u}}_{k}\left(\omega \right)$. Consider that the decomposition number is K. Through the optimization, the mode decomposition of $y_{usr}\left(m\right)$ can be expressed as

(18) $$y_{usr}\left(m\right)=\sum_{k=1}^{K}u_{k}\left(m\right)+r\left(m\right),$$

where $u_{k}\left(m\right)$ denotes the *k*-th IMF corresponding to $y_{usr}\left(m\right)$, and r(m) describes the residual.

After VMD, we use the Hilbert transform to obtain the corresponding analytic *k*-th IMF as

(19) $$z_{k}\left(m\right)=u_{k}\left(m\right)+j\hbar \left[u_{k}\left(m\right)\right],$$

where ℏ[·] denotes the Hilbert transform operation.

Note that the obtained IMFs have been upsampled and modulated. Hence, the final complex IMFs can be obtained by demodulation and downsampling. The demodulated results can be obtained by multiplying exponential factors $e^{-j2\pi \frac{m}{4}}$ as

(20) $$q_{k}\left(m\right)=z_{k}\left(m\right)e^{-j2\pi \frac{m}{4}}.$$

At last, we can acquire the final *k*-th complex BLIMF $x_{k}\left(m\right)$ corresponding to the origin complex data y(m) by downsampling as below

(21) $$x_{k}\left(m\right)=q_{k}\left(2m+1\right),0\leq m\leq M-1.$$

### 4.3. Model Fitting

After decomposition, we obtain a set of IMFs $x_{k}\left(m\right)$, where 0≤m≤M−1 and k=1,2,…K. These IMFs can correspond to the subsignals $s_{k}\exp\left(j\varphi_{m}\left(\theta_{k},r_{k}\right)\right)$ in Equation (13). Thus, we can estimate the $\theta_{k}$ and $r_{k}$ parameters by fitting the phase of $x_{k}\left(m\right)$ with the function $\varphi_{m}\left(\theta_{k},r_{k}\right)$. We can estimate the $s_{k}$ from the average amplitude of $x_{k}\left(m\right)$, 0≤m≤M−1.

The phase of the complex-valued signal $x_{k}\left(m\right)$ can be obtained by

(22) $$\hat{\varphi }\left(m\right)=\mathrm{Unwrap}\left\{\arctan\left(\frac{I\left[x_{k}\left(m\right)\right]}{R\left[x_{k}\left(m\right)\right]}\right)\right\},$$

where I[·] defines the imaginary part of the complex-valued data, and the Unwrap{·} operation represents the phase unwrapping algorithm. The applied unwrapping algorithm can be realized as: whenever the jump between successive phase angles is greater than or equal to *π* radians, we shift the phase angle by increasing an integer multiple of ±2π until the jump is less than π [31]. Then the calculation result $\hat{\varphi }\left(m\right)$ is fitted with the phase model in Equations (1) and (2) through the optimization:

(23) $$\underset{\theta_{k},r_{k}}{\min}\|\hat{\varphi }\left(m\right)-(\frac{2\pi }{\lambda }\left(\sqrt{r_{k}^{2}+m^{2}d^{2}-2mdr_{k}\sin\left(\theta_{k}\right)}-r_{k})\right){\|}_{2}^{2}.$$

This fitting problem can be easily solved by the nonlinear least square algorithms [32], and then the parameters $\theta_{k}$ and $r_{k}$ can be obtained. Furthermore, the source signal $s_{k}$ can be estimated by

(24) $$s_{k}=\frac{1}{M}\sum_{m=1}^{M}\left|x_{k}\left(m\right)\right|.$$

Finally, we obtain the *k*-th source’s location parameters: range $r_{k}$, direction $\theta_{k}$, and its amplitude $s_{k}$. We can classify the far-field and near-field sources from the value of $r_{k}$. If the $r_{k}$ is beyond $\frac{2D^{2}}{\lambda }$, the source is regarded as a far-field one. In that case, we only focus on the DOA of the source. Otherwise, the source is considered near-field, and both range and direction are required to describe the source’s position.

## 5. Results and Discussion

In this section, the performance of the CVMD-based source localization algorithm is validated in various conditions. The first part presents the scenarios for far-field sources DOA estimation. Then, we apply the proposed method to near-field sources localization and mixed near-field and far-field sources localization. In the last part of this Section, more performance of the CVMD-based method is investigated. The algorithms are implemented in MATLAB 2021a on a computer with an i7-8700 CPU and 8G of RAM to perform these simulations.

### 5.1. Far-Field Sources DOA Estimation

In this subsection for the far-field sources location examples, without a special note, the linear array of 20 sensors is uniformly distributed at half-wavelength. The divided grid of CBF, MVDR, MUSIC, and CS is set as [−90:0.1:90].

#### 5.1.1. Simulation 1

**Figure 2:**
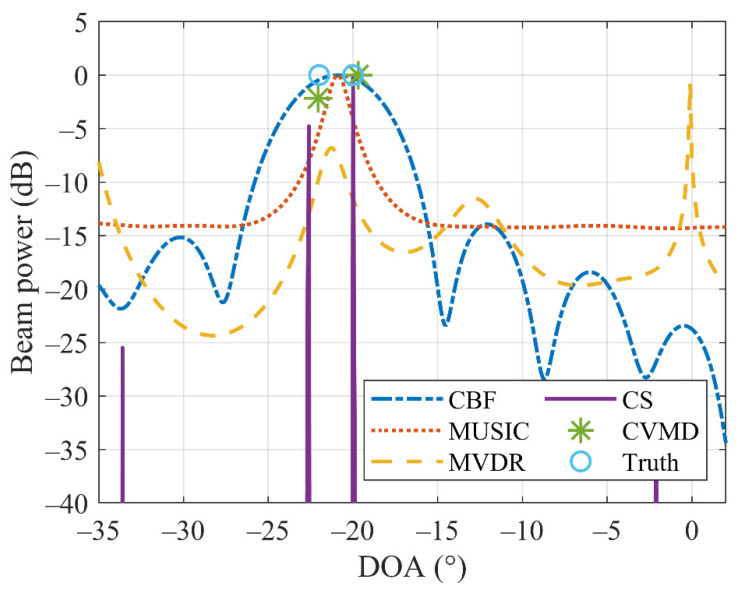
DOA estimation for two equal strength coherent sources at 20° and 22° by CBF, MUSIC, MVDR, CS, and CVMD method.

In this simulation, the resolution performance of the proposed method is investigated. The high resolution of DOA estimation is essential for distinguishing and locating adjacent sources. Assume that two coherent far-field sources from −22° and −20° with the same signal-to-noise ratio (SNR) = 20 dB are incident on the array. Figure 2 shows the beam power of the CBF (blue dash-dotted line), MUSIC (red dotted line), MVDR (yellow dashed line), CS (solid purple line), the proposed CVMD method (asterisk marker), and the true DOAs (light blue circle marker). It can be seen that CBF fails to discriminate between the two adjacent sources because of the limitation of the Rayleigh limit. MUSIC and MVDR also failed because of the condition of one snapshot and coherent sources. Despite CS having false targets and CVMD requiring prior knowledge about the number of sources, they retrieve the two sources successfully. However, the simulation running time of the CS-based method is more than four times longer than that of CVMD based method, which will be analyzed in Simulation 3.

This example shows that the proposed CVMD method performs well in distinguishing closely located sources. Moreover, it is suitable for the condition of single-snapshot and coherent sources.

#### 5.1.2. Simulation 2

**Figure 3:**
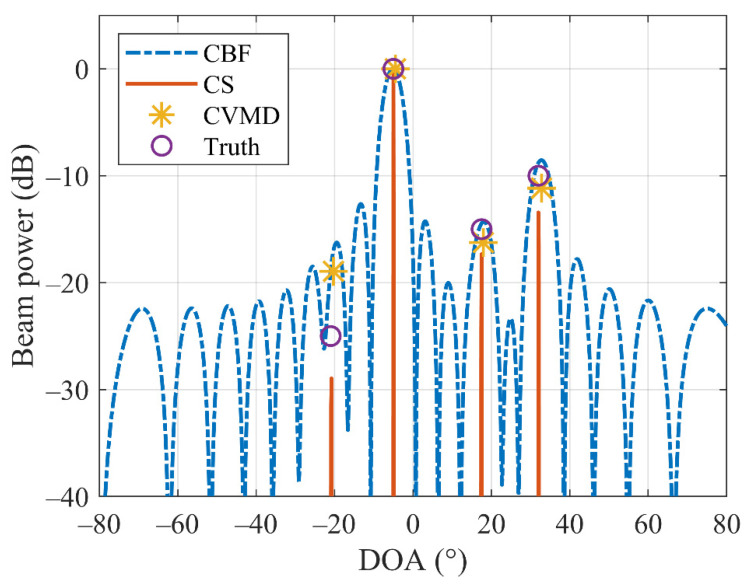
DOA estimation for four sources at −21° with SNR = 5 dB, −5° with SNR = 30 dB, 17.5° with SNR = 15 dB, and 32° with SNR = 20 dB by CBF, CS, and CVMD method.

In this simulation, the proposed method is verified to locate multiple sources and detect weak signals. It is often challenging to find weak sources because of the strong interference and insufficient data snapshots. Four far-field sources from −21° with SNR = 5 dB, −5° with SNR = 30 dB, 17.5° with SNR = 15 dB, and 32° with SNR = 20 dB are incident on the array. Since we still consider the condition on one-snapshot and coherent sources, the methods based on covariance matrices, such as MUSIC and MVDR, will not perform well. Therefore, we only compare our CVMD-based approach with CBF and CS. Figure 3 shows the DOA estimation by CBF, CS, and the proposed CVMD method. CBF fails to detect the weak sources at −21° and 17.5° successfully because of the strong sidelobes of the beam power. In contrast, CS and CVMD can locate all of the sources. However, the computing time of the CVMD-based algorithm only takes 0.45 s, while the CS-based method is almost 2.8 times that of the CVMD-based algorithm.

This example shows that the proposed method achieves great performance in locating multiple far-field sources, especially the weak sources in substantial interference, as that of the CS method. However, the computing time of the CVMD method is shorter than that of the CS method.

#### 5.1.3. Simulation 3

**Figure 4:**
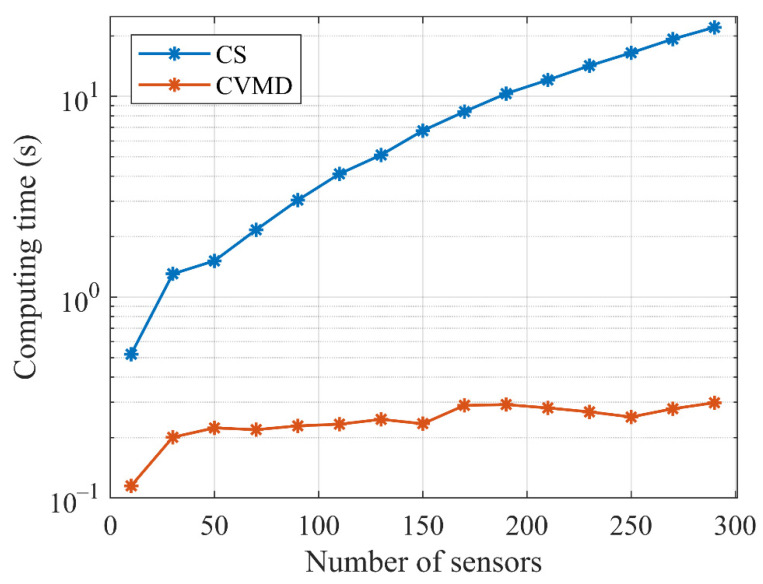
Comparison of computation time for two sources between the CS and CVMD-based methods with the different number of sensors.

**Figure 5:**
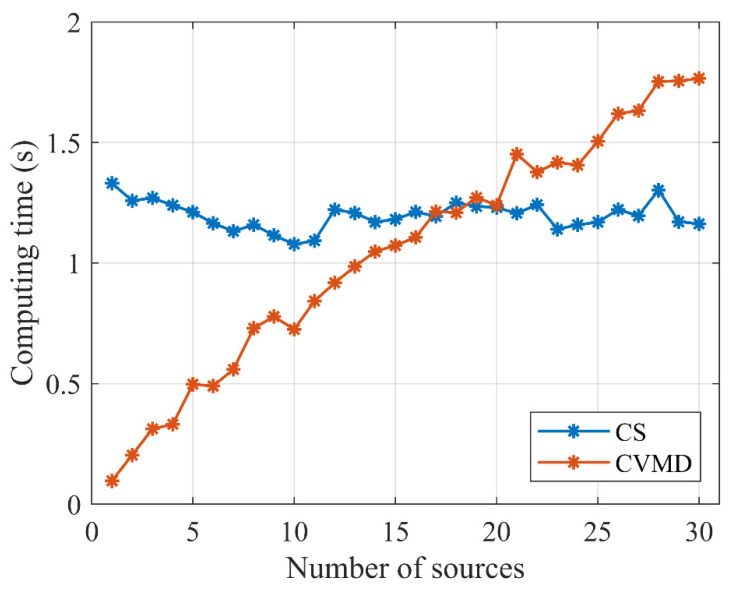
Comparison of computation time for 30 sensors between the CS and CVMD-based methods with the different number of sources.

In this subsection, we compare the computing time of the CS and CVMD method versus the number of sensors and the number of sources. The simulations were repeated 50 times independently, and then the consuming time of CS and CVMD were averaged, respectively. Figure 4 shows the computing time towards different numbers of array elements with two incoming sources. It can be seen that the calculation time of the CS method (blue lines) increases almost exponentially with the increase in the number of sensors. For the proposed CVMD method (red lines), the calculation time is practically independent of the number of array elements. On the whole, the computing time of the CVMD method is much lower than that of the CS method in all of the cases. We also investigate the consumption time of the two methods for 30 sensors versus the number of the sources, as shown in Figure 5. It can be seen that the calculation time of the CVMD method (red lines) increases almost linearly with the increase in the number of sources. Moreover, the consuming time of the CS method (blue lines) does not relate to the number of sources. When the number of sources is between 1 and 16, the CVMD-based method has a faster run speed than the CS-based method. When the number is around 17 to 20, the consumption time of the two ways is almost the same. When the number of sources exceeds 20, the calculation time of the CS-based method is shorter than that of the CVMD method. In practical application, the number of sources to be estimated will not be huge. In contrast, extensive arrays with a great number of sensors have been a trend nowadays. 

In conclusion, the computational complexity of the CVMD-based method only relates to the number of sources independent of the number of sensors and has a faster run-speed than the CS-based method in the few source numbers and large array cases, which will show advantages in practical application for real-time tracking and localization.

### 5.2. Near-Field Sources DOA Estimation

#### 5.2.1. Simulation 4

**Figure 6:**
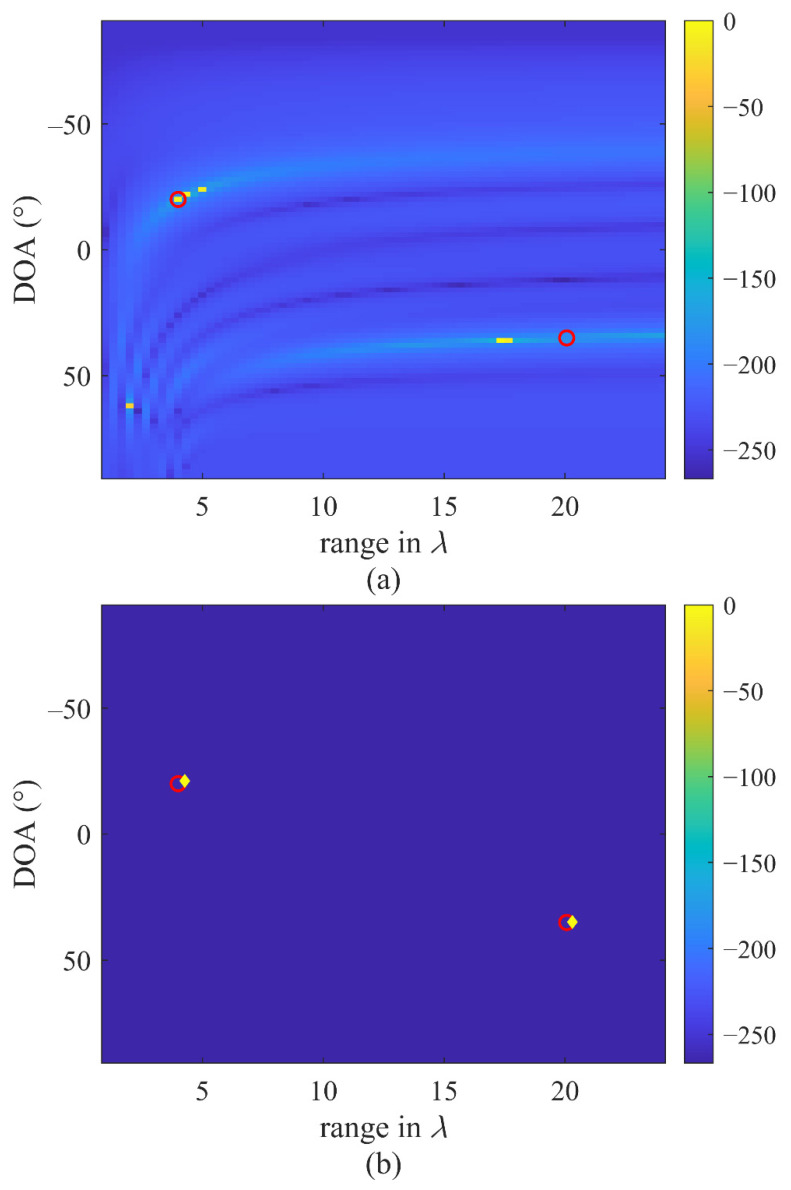
Two near-field sources localization results and the actual positions (red circle): (**a**) The power estimation at the grid of DOA and range by the CS-based method, (**b**) The DOA and range estimation by the CVMD-based method (yellow rhombus).

In this simulation, we suppose a near-field scenario to prove the proposed method’s ability for near-field sources localization. The number of the array elements is set as 20, and the spacing is set as λ/4. Two equal strength sources located at DOA and range of (−20°, 4λ*λ*) and (35°, 20λ+1) with SNR = 20 dB are incident on the array. We compare the CS method and the CVMD method in estimating the DOA and range. The grid of direction and range for the CS method is set as [−90:2:90] and [λ:λ/3:24λ]. Thus, the first source is on the grid, and the second source is off-grid. Figure 6a,b show the localization results by CS and the CVMD method. It can be seen that the CS method can locate the on-grid source but fails to locate the off-grid source correctly, especially in the range dimension. In contrast, the CVMD method does not need a grid, and both two sources are located successfully.

This simulation proves the ability of the proposed method in near-field source localization. Notably, the CVMD-based method does not need the grid. Thus, the estimation accuracy is not affected by the grid accuracy like the CS method.

#### 5.2.2. Simulation 5

**Figure 7:**
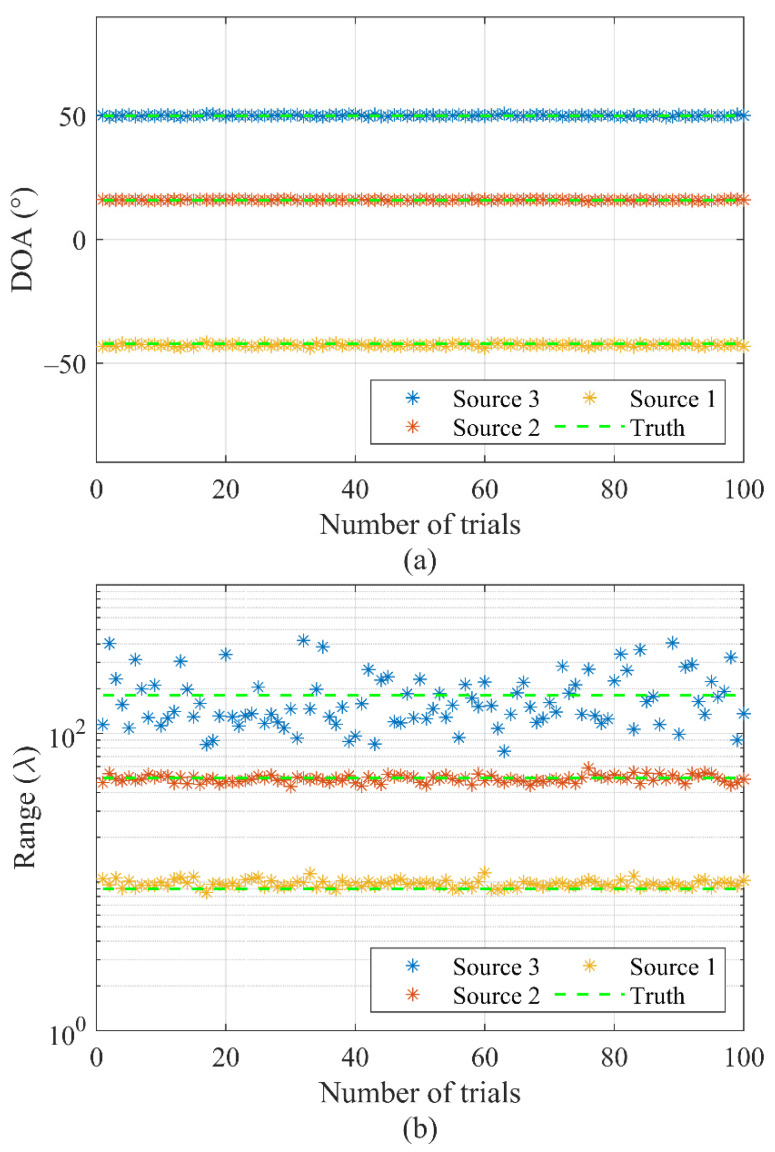
Localization for two near-field sources and one far-field source of 100 independent trials by the CVMD-based method: (**a**) DOA estimation. (**b**) Range estimation.

This subsection considers the mixed far-field and near-field sources scenario, which is more common in the real world. Two near-field sources located at DOA and range of (−42°, 9λ) and (16°, 50λ) and a far-field source located at (50°, 180λ) are incidents on the array of 20 elements. Figure 7a,b show the DOA and range estimation by the CVMD method of 100 independent trials, respectively. It can be seen that all of the trials yield accurate DOA estimations. For near-field sources, the estimates of the range are almost accurate, whereas, for the far-field source, they are of high variance. Since the goal for far-field sources is only to estimate the DOA, the range can be ignored as long as it can be separated from the near-field sources.

This simulation demonstrates that the proposed source localization algorithm deals well with the mixed near-filed and far-field sources situation. Furthermore, the far-field and near-field sources can be distinguished and classified from the estimated ranges.

#### 5.2.3. Simulation 6

In this simulation, we compare the computational burden of the CVMD-based method and the CS-based method for locating mixed near-filed and far-filed sources. The computing time of each method is calculated by averaging 50 independent trials. The source number is set as two, and the sensor number is set as 30. For the CS-based method, the calculation time is closely related to the size of the grid. Since both range and direction are required, the grid becomes more complex, and the time consuming becomes larger than in simulation 3. Suppose simulation A sets the angled grid as [−90:2:90] and the range grid as [0:λ:200λ], then the size of the grid is [91×200]. In this case, the average consuming time of the CS-based method is 15.8 s, while the CVMD-based method takes only 0.19 s. If we increase the resolution of the angled grid to [−90:1:90] in Simulation B, the computing time of the CS-based method will grow to 37.6 s. Similarly, the run time will increase to 50.05 s when the range grid becomes [0:λ:500λ] in Simulation C. Table 1 lists the run time of the CVMD-based method and the CS-based method for the CS grid size [91×200], [181×200], and [91×500]. It can be seen that the CVMD-based method needs much lower computation time than the CS-based method. Moreover, the proposed method is not limited to the grid. Specifically, no matter how far the sources of interest are, the CVMD-based method keeps low computing time.

### 5.3. Performance Analysis

#### 5.3.1. Simulation 7

**Figure 8:**
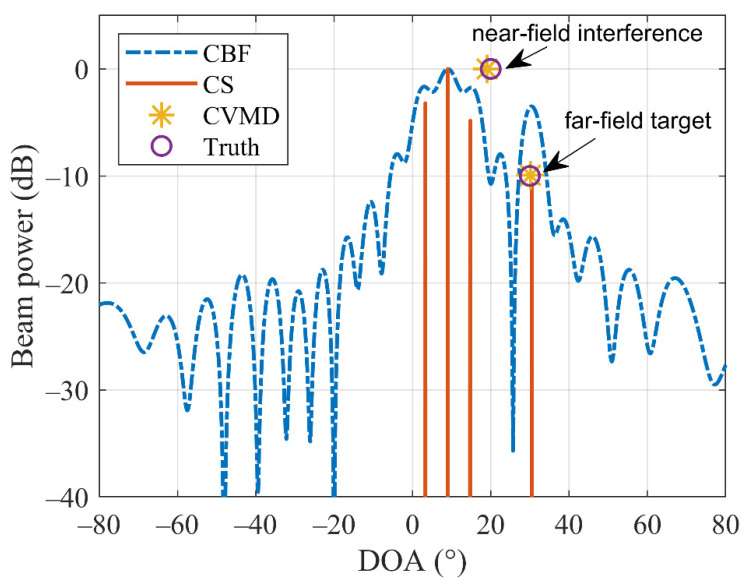
Far-field source DOA estimation in the presence of near-field interference by CBF, CS-based method, and CVMD-based method.

In practice, interference is often inevitable when locating the target source, which will easily cause false targets. In this simulation, we investigate the performance of our CVMD-method on far-field source DOA estimation in the presence of near-field interference. Suppose the far-field source is at 30°, the near-field interference locates at DOA and range of (20°, 25λ), the number of the array elements is set as 20, and the spacing is set as λ/2. Figure 8 shows the DOA estimation results by CBF, CS, and CVMD. It can be seen that the CBF and CS methods have false targets due to the near-field interference, which is misleading. On the contrary, the CVMD-based approach is robust with interference. It gives the correct DOA estimation of the far-field target without introducing false targets.

#### 5.3.2. Simulation 8

In this simulation, we investigate the impact of the source signal’s propagation attenuation on the performance of our CVMD-method. Suppose a source from (20°, 10λ) impinge on the array with 20 elements, the element spacing is set as λ/4, and the SNR is set as 20. For the spherical wave, the signal attenuates according to the square of the distance in the process of propagation. If we consider the attenuation coefficient, the signal model in Equation (1) can be rewritten as

(25) $$y_{m}=\sum_{k=1}^{K}\frac{s_{k}}{r_{m,k}^{2}}\exp\left(\frac{j2\pi }{\lambda }\left(r_{m,k}-r_{k}\right)\right)+n_{m},$$

To investigate whether the propagation attenuation affects the location results, we first apply the CVMD-based method to the simulation data without considering propagation attenuation as Equation (1). Then, we apply the method to the data generated as Equation (25). The simulation is repeated 200 times independently. Table 2 shows the root mean square error (RMSE) of the DOA and range estimation by the CVMD method towards the data without propagation attenuation and with propagation attenuation. It can be seen that the RMSEs are almost the same. Therefore, it can be concluded that the CVMD-based method is robust with the propagation attenuation of the source signal.

## 6. Conclusions

This paper presents a near-field and far-field source localization algorithm with a uniform linear array. The algorithm is motivated by the novel time-domain nonstationary signal processing method named complex variational mode decomposition (CVMD). The CVMD algorithm provides a different solution to inverse problems. Hence, we extend the CVMD method to the source localization problem to yield great performance with low computation.

Serval experiments verify the effectiveness of the proposed algorithm. Compared with the traditional localization techniques based on the data covariance matrix, the proposed method shows advantages in dealing with coherent sources and the snapshots absence condition. Compared with the methods based on sparse signal recovery, such as compressive sensing, our proposed algorithm requires much lower computing time when yielding a similar localization performance on resolution, detection, and classification of near and far-field sources.

The shortcoming is that our method requires the number of sources to be predefined. Many successful algorithms, such as MUSIC, suffer from the same drawback. In practical situations, we usually have some prior information. Moreover, there are many preprocessing techniques to estimate the number of sources, which will help us improve the current method. Another shortcoming is that the decomposition may have multiple solutions. This problem can also be improved by prior information.

Furthermore, extending the CVMD from the time domain to the spatial domain brings benefits not only to source localization but also to other array signal-processing purposes. For example, we can use the CVMD for near-field noise suppression because the method can decompose the array measurements into several subsignals corresponding to the potential sources at different locations. Thus, we can extend the proposed CVMD-based method to more array signal processing applications in the future.

## Figures and Tables

**Table 1:** Comparison of the computing time.

	Simulation A Grid [91×200]	Simulation B Grid [181×200]	Simulation C Grid [91×500]
CS	15.8 s	37.6 s	50.05 s
CVMD	0.19 s	0.20 s	0.23 s

**Table 2:** The RMSE of the DOA and range estimation by the CVMD-based method.

	DOA	Range
Without propagation attenuation	0.241°	0.125*λ*
With propagation attenuation	0.236°	0.126*λ*

## Data Availability

The data that support the findings of this study are available from the corresponding author upon reasonable request.
